# PyHLA: tests for the association between HLA alleles and diseases

**DOI:** 10.1186/s12859-017-1496-0

**Published:** 2017-02-06

**Authors:** Yanhui Fan, You-Qiang Song

**Affiliations:** 1 School of Biomedical Sciences, The University of Hong Kong, 21 Sassoon Road, Pokfulam, Hong Kong Hong Kong; 2 Centre for Genomic Sciences, The University of Hong Kong, 5 Sassoon Road, Pokfulam, Hong Kong Hong Kong; 3 Department of Cancer Genomics, LemonData Biotech (Shenzhen) Ltd., Shenzhen, China

**Keywords:** HLA, Association, Interaction, Multi-allelic

## Abstract

**Background:**

Recently, several tools have been designed for human leukocyte antigen (HLA) typing using single nucleotide polymorphism (SNP) array and next-generation sequencing (NGS) data. These tools provide high-throughput and cost-effective approaches for identifying HLA types. Therefore, tools for downstream association analysis are highly desirable. Although several tools have been designed for multi-allelic marker association analysis, they were designed only for microsatellite markers and do not scale well with increasing data volumes, or they were designed for large-scale data but provided a limited number of tests.

**Results:**

We have developed a Python package called PyHLA, which implements several methods for HLA association analysis, to fill the gap. PyHLA is a tailor-made, easy to use, and flexible tool designed specifically for the association analysis of the HLA types imputed from genome-wide genotyping and NGS data. PyHLA provides functions for association analysis, zygosity tests, and interaction tests between HLA alleles and diseases. Monte Carlo permutation and several methods for multiple testing corrections have also been implemented.

**Conclusions:**

PyHLA provides a convenient and powerful tool for HLA analysis. Existing methods have been integrated and desired methods have been added in PyHLA. Furthermore, PyHLA is applicable to small and large sample sizes and can finish the analysis in a timely manner on a personal computer with different platforms. PyHLA is implemented in Python. PyHLA is a free, open source software distributed under the GPLv2 license. The source code, tutorial, and examples are available at https://github.com/felixfan/PyHLA.

## Background

The human leukocyte antigen (HLA) loci on chromosome 6 (6p21.3) are the most polymorphic and gene-dense region of the human genome. HLA proteins play an important role in transplant rejection. Association of variants in the HLA region and infectious, autoimmune diseases and cancers has been established. Directly typing HLA using experiments is still laborious, expensive, and time-consuming [1]. Several algorithms and pipelines, such as HLA*IMP:02 [2] and MGAPrediction [3] have been developed for HLA imputation using data from genome-wide association studies (GWAS), whereas OptiType [4], HLA-VBSeq [5] and HLAreporter [6] have been developed for HLA typing using data from next-generation sequencing (NGS) studies. All tools use HLA allele sequences from the IMGT/HLA database [7] as reference. These tools have provided us a cost-efficient, high-throughput approach for HLA typing by using the already available GWAS and NGS data.

Given the continuously increasing amounts of HLA types being generated, integrating the workflow for their downstream association analysis is highly desirable. Several existing tools, such as CLUMP [8], PyPop [9] and SKDM [10], can be used to analyze HLA types. These tools are not ideal for association analysis of HLA types inferred from GWAS and NGS data as they were designed for analyzing microsatellite markers or provided limited functions. In this study, we present PyHLA, a Python-based standalone tool, for the association analysis between diseases and HLA types inferred from GWAS and NGS data.

## Implementation

**Fig. 1:**
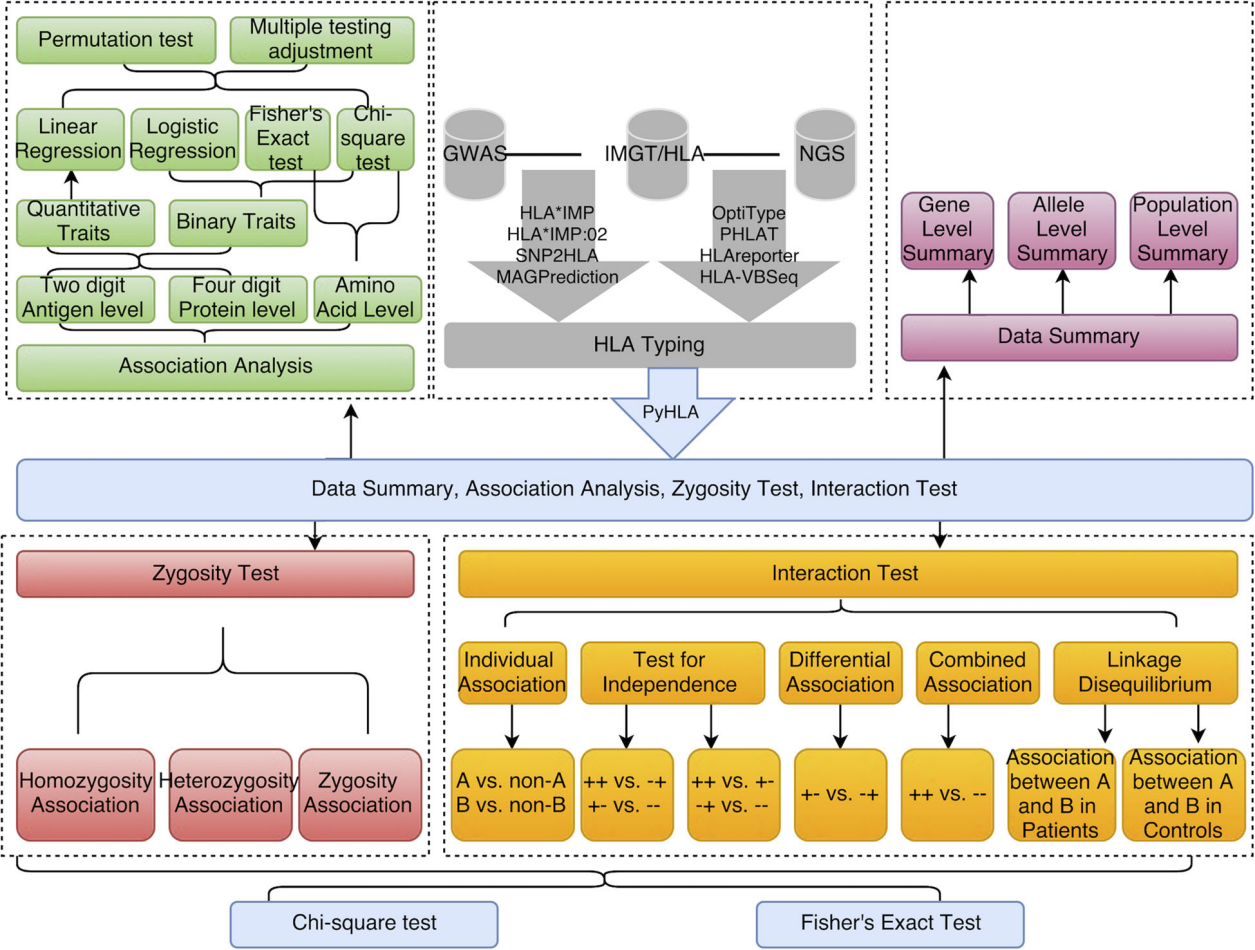
PyHLA flowchart. PyHLA uses the imputed HLA types from several softwares (*top-middle*) as input. Four main functional modules of PyHLA are: data summary (*top-right*), association analysis (*top-left*), zygosity test (*bottom-left*) and interaction test (*bottom-right*). For details of the manner in which each module works, please see https://github.com/felixfan/PyHLA

PyHLA is implemented in Python 2.7. The graphical user interface is also provided. The source code, tutorial and examples are freely available at https://github.com/felixfan/PyHLA. A demonstration is also available at https://github.com/felixfan/PyHLA/tree/master/demo. Figure 1 shows an overview of the methods applied to HLA types for finding disease-associated HLA alleles.

### Data summary (module 1)

Gene, allele and population level summary of the frequency can be produced in the case and control populations.

### Association analysis (module 2)

It is a simple and easy way to implement methods for localization of susceptibility genes by comparing the allele frequencies between cases and controls from the same population. Usually, Pearson’s chi-squared test or Fisher’s exact test is performed on a 2 × 2 contingency table, which contains the counts of minor and major alleles for a single locus in cases and controls. As the most polymorphic part of the human genome, HLA genes, such as HLA-A, HLA-B and HLA-C, have several thousand known alleles [7]. PyHLA performs Pearson’s chi-squared test or Fisher’s exact test on the 2 × 2 contingency table, which compares one allele with the other alleles grouped together.

If the HLA-A gene has *n* common alleles in cases and controls, then *n* tests are performed. In each test, one allele is compared with the other *n* − 1 alleles grouped together. The allelic 2 × 2 contingency table for a specific HLA allele contains the counts of this allele and the counts of other *n* − 1 alleles in cases and controls. The dominant and recessive models assume that each allele is dominant and recessive to the other *n* − 1 alleles, respectively. The dominant 2 × 2 contingency table for a specific HLA allele contains the counts of individuals with and without the allele in cases and controls. The recessive 2 × 2 contingency table for a specific HLA allele contains the counts of individuals with and without two copies of this allele in cases and controls.

### Pearson’s Chi-squared test

Pearson’s chi-squared test statistic can be calculated using the following formula:

1 $$ {\chi}^2=\kern0.5em {\displaystyle {\sum}_{i=1}^r{\displaystyle {\sum}_{j=1}^c\frac{{\left({O}_{i, j}-{E}_{i, j}\right)}^2}{E_{i, j}}}}, $$

where *χ* ^*2*^ is the chi-square critical value with a degree of freedom equals to 1. *O*_*i,j*_ and *E*_*i,j*_ are the observed and expected frequencies of the cell in row *i* and column *j*, respectively. *r* is the number of rows and *c* is the number of columns; both are 2 for the 2 × 2 contingency table.

### Fisher’s exact test

Fisher’s exact test first calculates the exact probability of the 2 × 2 contingency table of the observed values using the following formula:

2 $$ {P}_{c utoff}=\frac{r_1!{r}_2!{c}_1!{c}_2!}{N!{\displaystyle {\prod}_{i, j}}{O}_{i, j}!}, $$

where *O*_*i,j*_ is the observed frequency of the cell in row *i* and column *j. r*_*i*_ and *c*_*i*_ are the rows and columns of marginal totals, respectively. *N* is the grand total. *P*_*cutoff*_ is the exact probability of obtaining such set of observed values. Then, the probability for all possible tables with the same marginal totals is calculated. The two-sided *p* value for the Fisher’s exact test is calculated by summing all probabilities less than or equal to *P*_*cutoff*_.

### Logistic and linear regression

Logistic and linear regressions were also implemented for disease trait and quantitative trait, respectively. These two regression methods allow for multiple covariates when testing for allele and amino acid (AA) association. The covariates can be either continuous or binary. A genotype will be coded as 0, 1, or 2, depending on the number of effect allele it carries and the tested genetic model (Table 1).

**Table 1:** Genotype coding for additive, dominant, and recessive models, with D being the risk allele

Genotype	Code		
	Additive	Dominant^a^	Recessive^b^
DD	2	1	1
Dd	1	1	0
dd	0	0	0

^a^D is dominant over d

^b^D is recessive to d

The logistic regression without additional covariates is defined by the following formulas:

3 $$ \theta (x) = \Pr \left\{ y=1\Big| x\right\}, $$

4 $$ log\frac{\theta (x)}{1-\theta (x)}={\beta}_0+{\beta}_1 x, $$

where *y* is the binary outcome. 1 and 0 represent the disease and normal, respectively. *x* is the codes of genotypes. *β*_0_ is the constant term, and *β*_1_ is the coefficient of *x*. When extra covariates was added, the logistic regression is extended as follows:

5 $$ \theta (x) = \Pr \left\{ y=1\Big| x, co{v}_1, co{v}_2,\cdots co{v}_k\right\}, $$

6 $$ log\frac{\theta (x)}{1-\theta (x)}={\beta}_0+{\beta}_1 x + {\beta}_2 c o{v}_1 + {\beta}_3 c o{v}_2 + \cdots + {\beta}_{k+1} c o{v}_k, $$

where *cov*_*k*_ is the *k*th covariate and *β*_*k*+1_ is the coefficient of the *k*th covariate.

The simple linear regression with one dependent variable and one independent variable is defined by the following formula:

7 $$ y = {\beta}_0 + {\beta}_1 x + \varepsilon, $$

where *y* is the dependent variable, *x* is the independent variable, *β*_0_ is the constant term, *β*_1_ is the coefficient of *x*, and *ε* is the error term. The ordinary least squares method was used to estimate the parameters. When one or multiple covariates are added to the model, the linear regression model is defined by the following formula:

8 $$ y = {\beta}_0 + {\beta}_1 x+{\beta}_2 c o{v}_1+{\beta}_3 c o{v}_2+\cdots +{\beta}_{k+1} c o{v}_k + \varepsilon, $$

where *cov*_*k*_ is the *k*th covariate and *β*_*k*+1_ is the coefficient of the *k*th covariate.

### Multiple testing correction

The *p* values can be adjusted by using the Bonferroni correction or false discovery rate (FDR) correction. The empirical *p*-values can also be calculated using a permutation test, which randomly shuffles the phenotypes for individuals, while keeping the HLA alleles unchanged.

### Amino acid association analysis

PyHLA can perform not only allele level association analysis but also the AA level association analysis. The aligned AA sequences were retrieved from the IMGT/HLA database [7]. Fisher’s exact test or Pearson’s chi-squared test can be conducted to investigate AA occurrence that are significantly associated with a disease.

### Zygosity test (module 3)

Three tests were performed to investigate homozygous, heterozygous, and zygosity associations. These three tests evaluate the frequency difference of subjects carrying the homozygous and heterozygous alleles and the absence of a particular allele/AA in cases and controls. An individual carrying two same alleles is considered homozygous in the allele level test. An individual carrying two identical alleles or an individual carrying two different alleles that code for the same AA residue is considered homozygous in the AA level test. Fisher’s exact test or Pearson’s chi-squared test for a 2 × 2 contingency table can be used for the zygosity test.

### Interaction test (module 4)

Interaction test performs eight tests for detecting the strongest association. These tests involve tests for independence, differential association, combined association, linkage disequilibrium, and interaction [10, 11]. Each of the eight tests is based on a 2 × 2 contingency table. Fisher’s exact test or Pearson’s chi-squared test can be used for the interaction test.

## Results

Since it is hard to find a publicly available real dataset. A simulated data set with 1000 cases and 1000 controls was used to demonstrate the usage of PyHLA. Detailed commands, inputs and outputs are available on https://github.com/felixfan/PyHLA/tree/master/demo.

Association test suggested that the two most significant alleles are HLA-A*01:01 (*P* = 4.03E-24, OR = 2.15) and HLA-DQB1*05:02 (*P* = 3.32E-11, OR = 1.58). Zygosity test further showed that the susceptibility to disease between homozygote and heterozygote of these two alleles are different (*P* = 2.46E-11 and *P* = 1.10E-7 for HLA-A*01:01 and HLA-DQB1*05:02, respectively.). The heterozygotes are individually associated with the disease (*P* = 1.21E-19 and *P* = 6.60E-8 for HLA-A*01:01 and HLA-DQB1*05:02, respectively.). Finally, the interaction test suggested that HLA-A*01:01 and HLA-DQB1*05:02 are in linkage disequilibrium in cases; their combined action is contributory to disease susceptibility.

## Discussion

PyHLA provides an integrated pipeline for detecting HLA association in antigen (two-digit allele level), protein (four-digit allele level) and AA levels. Zygosity tests will examine the homozygous, heterozygous, and zygosity associations once the associated alleles and AAs are identified. In addition, interaction test examines the independence, differential association, combined association, interaction, and linkage disequilibrium between two factors.

In addition to identifying alleles and AA residues that are significantly associated with the disease, PyHLA also tests whether the increased HLA homozygosity or heterozygosity contributes to the increased susceptibility to a disease. When several factors are associated with the disease, the interaction test identifies the strongest one between each pair of the two factors. The factor with the strongest association is more likely to be the causative factor that truly contributed to the disease [11].

In this work, Pearson’s chi-squared test and Fisher’s exact test performed on a 2 × 2 contingency table were implemented in PyHLA. Linear and logistic regressions were also included to consider multiple covariates simultaneously.

Bonferroni adjustment and correction via FDR estimation are widely used for multiple testing corrections. Bonferroni correction assumes that all tests are independent and is conservative in genetic association analysis, whereas FDR is less stringent [12–15]. In addition, the empirical *p* values can also be calculated using the permutation test, which randomly shuffle the phenotypes for individuals while keeping the HLA alleles unchanged. The permutation test preserves the correlation structure among HLA alleles but requires a large number of random shuffles. Given that the number of HLA alleles is relatively smaller than the number of SNPs in the genome, the computing time and resources needed for the permutation test are significantly less. PyHLA can perform these analyses on a single modern personal computer in a timely manner.

Four chi-squared tests were implemented in CLUMP [8] to test the association between disease and alleles at highly polymorphic loci, and Monte Carlo imputation was performed to estimate the significance level. CLUMP is mainly designed for analyzing microsatellite markers in qualitative trait studies (case-control study), but not in quantitative trait studies. CLUMP cannot perform residual level tests as well. SKDM [10] is specialized in case-control HLA analysis through the identification and subsequent dissection of AA association; it is not designed for quantitative studies. Only the Fisher’s exact test is available for association test, and only Bonferroni correction is available for multiple testing adjustment. PyPop [9] is designed to handle large sample sizes for population statistics, haplotype frequency estimation and linkage disequilibrium significance testing. PyHLA is designed to supplement and extend these existing software. PyHLA can handle both qualitative and quantitative trait studies in both amino acid level and different resolutions of allele levels. Both chi-squared test and Fisher's exact test are implemented to test the association, and both Bonferroni correction and FDR are available for multiple testing adjustment. Monte Carlo imputation is also implemented to estimate the significance level. Moreover, logistic regression and linear regression implemented in PyHLA can also include covariates in the association analysis.

## Conclusions

In summary, PyHLA is a user-friendly tool for HLA association analysis. Existing methods are integrated and additional desired methods are included in PyHLA. PyHLA is applicable to small and large sample sizes and can complete the analysis in a timely manner on a personal computer. PyHLA is designed for case-control studies. PyHLA is currently unable to analyze family-base datasets.

## Abbreviations

AA: Amino acid
FDR: False discovery rate
GWAS: Genome-wide association study
HLA: Human leukocyte antigen
NGS: Next-generation sequencing
